# *Morinda officinalis* oligosaccharides alleviate chronic unpredictable mild stress-induced depression through the BDNF/TrkB/CREB pathway and symptoms of sexual dysfunction in mice

**DOI:** 10.3389/fnins.2024.1509543

**Published:** 2025-01-08

**Authors:** Mengjie He, Mengying Hu, Tingqiao Wang, Zeping Zuo, Hongkai Li, Zhiwei Zhao, Yunwen Hao, Xueling Dai, Jianfang Wang, Yaxuan Sun

**Affiliations:** ^1^Beijing Key Laboratory of Bioactive Substances and Functional Food, Beijing Union University, Beijing, China; ^2^School of Chinese Materia Medica, Beijing University of Chinese Medicine, Beijing, China; ^3^Beijing Tongrentang Company Limited, Beijing, China, China; ^4^Faculty of Land and Food Systems, University of British Columbia, Vancouver, BC, Canada; ^5^Dongfang Hospital, Beijing University of Chinese Medicine, Beijing, China

**Keywords:** *Morinda officinalis* oligosaccharides, depression, CUMS, BDNF/TrkB/CREB pathway, gut microbiota, sexual dysfunction

## Abstract

**Background:**

In recent years, depression has become a global public health concern, and one of the common concomitant symptoms are diminished sexual motivation and impaired sexual performance. The aim of this study was to investigate the potential effects of *Morinda officinalis* oligosaccharides (MOO) on depression and its concomitant symptom, sexual dysfunction.

**Methods:**

Chronic unpredictable mild stress (CUMS)-induced depression model was constructed, and the effects of MOO on depression and sexual abilities were evaluated.

**Results:**

The results revealed that MOO was able to alleviate CUMS-induced depression-like behavior in mice, to inhibit hippocampal neuron apoptosis, to reverse monoamine neurotransmitter imbalance, increase Brain-derived neurotrophic factor (BDNF) expression levels in the hippocampus, to modulate the composition and distribution of gut microbiota, and to increase the abundance of probiotics after continuous gavage of MOO for 28 days. MOO further confirmed that sexual dysfunction is closely related to the development of depression by improving the lack of sexual motivation and low sexual performance in CUMS-induced depressed mice, modulating the disruption of sex hormone secretion in serum, and alleviating sperm morphology and functional defects in the epididymis.

**Conclusion:**

These findings on MOO provide a basis for exploring its antidepressant mechanism, its use to improve hypogonadotropic symptoms, and for future development of new antidepressant drug to improves hypogonadotropic symptoms.

## 1 Introduction

Stressful stimuli are aplenty in modern society, and long-term chronic stressful stimuli are an important cause of depression, which affects approximately 300 million people worldwide and has become a serious public health problem and has attracted widespread attention (Wang W. et al., 2024). People with depression not only exhibit behavioral and mood disorders but also changes in brain structure and dysfunction (Zhang Y. et al., 2024b). This leads to fatigue, endocrine disorders, as well as altered sexual performance, impairing memory, concentration, and judgment (Kim et al., 2024). Another great concern frequently overlooked is that sexual dysfunction, characterized by diminished sexual motivation and impaired sexual performance, is a major comorbidity in depressed patients. Depressed male patients suffering from chronic stress tend to exhibit reduced sexual interest, difficulty with sexual arousal, and poor sexual function (Wang Z. et al., 2021). This co-morbidity not only exacerbates the difficulty of treatment but also the economic burden on patients. Currently, there are few drugs that can co-treat depression and sexual dysfunction in clinical practice. Moreover, numerous experimental and clinical studies have found that commonly used antidepressant drugs exhibit side effects such as slow onset of action, low cure rates, and sexual dysfunction in practice (Peng et al., 2024). Therefore, there is an urgent need to find drugs that can clinically improve emotional dysfunctions without disrupting sexual activity. Traditional Chinese medicine is characterized by holistic regulation, and herbal medicine has attracted much attention from researchers for its therapeutic advantages of multi-targets, multi-pathways, and multi-systems.

**Figure 1:**
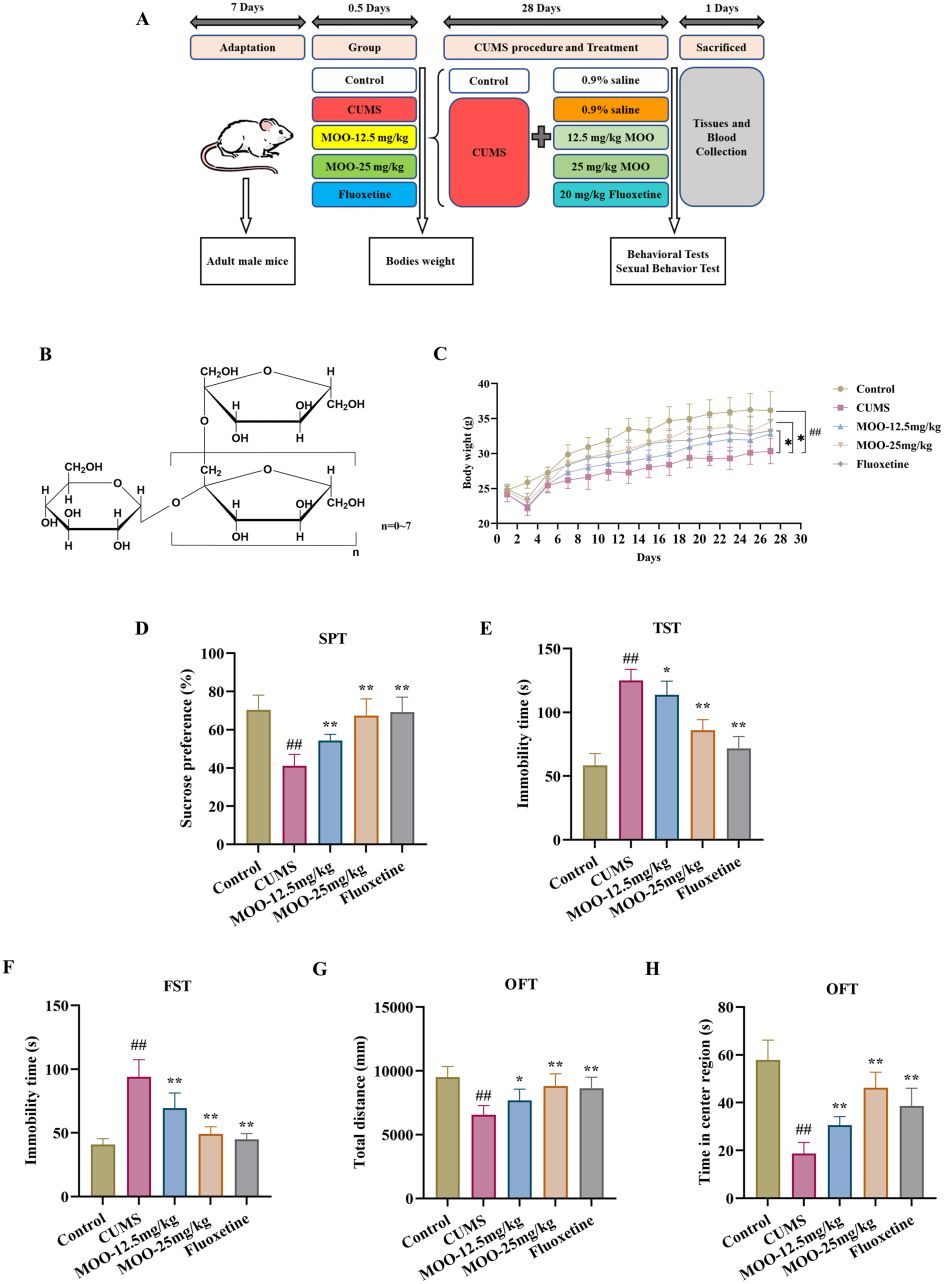
MOO improved depression-like behavior in CUMS-Induced mice. **(A)** Timeline of CUMS procedure, drug administration, behavioral tests, mouse sacrifice, tissue and blood collection. **(B)** The general formula of the structure of MOO; **(C)** 28-day mouse body weight; **(D–H)** Behavioral experiments include SPT **(D)**, TST **(E)**, FST **(F)**, and OFT **(G–H)** to assess depressive-like behavior in CUMS mice. Results were expressed as mean ± SD (*n* = 10). ^##^*p* < 0.01 vs. control group; ^*^p < 0.05 and ^**^p < 0.01 vs. CUMS group.

*Morinda officinalis* (*Morinda officinalis How*.) is a medicinal plant of the genus *Morinda officinalis* in the family Rubiaceae, and the main medicinal part is the root (Chen et al., 2023). It is mainly distributed in the tropical and subtropical regions of southern China, including Fujian, Guangdong, Hainan, and Guangxi (Jiang et al., 2024). Modern pharmacological studies have reported a variety of bioactivities associated with *Morinda officinalis*, such as protecting the liver (Zhu et al., 2024) and strengthening the bones (Cao et al., 2024). *Morinda officinalis* oligosaccharides (MOO) are a natural extract from the root of *Morinda officinalis*, which consist of 2–9 monosaccharides and are hardly absorbed by the human body. The general formula of the structure of MOO is shown in Figure 1B. As the main active ingredient in *Morinda officinalis*, it has a wide range of pharmacological effects, including antioxidant (Cha et al., 2019), anti-inflammatory (Lai et al., 2023), anti-stress (Li et al., 2001), and improving Alzheimer’s disease (Deng et al., 2020). In addition, MOO has been shown to have antidepressant effects (Yang et al., 2023), protect human sperm DNA from H_2_O_2_ damage, and enhance reproductive function (Chen et al., 2014). However, it remains unknown whether MOO could ameliorate chronic stress-induced impairments of sexual activity. In order to further investigate whether the antidepressant effect of MOO involves the regulation of sexual activity, the present study explored the effects of MOO on symptoms related to chronic stress-induced depression and sexual dysfunction in male mice from multiple perspectives based on the chronic unpredictable mild stress (CUMS) model, aiming to provide data support for the clinical prevention or treatment of depression and sexual dysfunction with MOO.

## 2 Materials and methods

### 2.1 Animals

Specific pathogen-free grade ICR mice (male, 8 weeks old, 18–22 g), 50 mice, were purchased from Beijing Viton Lever Charles River Animal Co. Ltd. [License No. SCXK (Jing) 2021-0006] and housed in the SPF-grade animal room of the Health Food Functional Testing Centre of the College of Applied Arts and Sciences, Beijing Union University [License No. SYXK (Jing) 2017-0038], with a rearing temperature of 22–24°C, a humidity of 45%, and a day/night cycle of 12 h (lighting time, 08:00–20:00). The mice were acclimatized for 7 days prior to performing the experiment, with free water and normal food for the duration of the experiment. All procedures in this experiment were performed in accordance with the Regulations of the Chinese Council on Animal Care and approved by the Health Food Functional Testing Centre of the College of Applied Arts and Sciences of Beijing Union University (JCZX11-2309-1).

### 2.2 CUMS modeling

The CUMS procedure was carried out as previously described by Xie et al. (Xie M. et al., 2024), with minor modifications: 12 h of food fasting; 12 h of water fasting; 24 h of moist bedding; 4 h of restraint stress; 24 h of cage tilting at 45°C; 5 min of swimming in warm water at 45°C; and 12 h of blinking throughout the night; suspension of the tail for 5 min (1 cm from the tail end); electroshock (2 mA, 4 s, 10 s intervals, repeated 2 times). Mice were subjected to different types of stimuli at different times of the day. The stimulation procedure was not repeated on both days, and the mice were unable to predict the onset of stress. At the end of the acclimatization feeding, the CUMS procedure was initiated.

### 2.3 Animal grouping and drug administration

The animals were randomly divided into five groups (*n* = 10): control group (control); CUMS + saline group (CUMS); CUMS + 12.5 mg/kg MOO group (MOO-12.5 mg/kg); CUMS + 25 mg/kg MOO group (MOO-25 mg/kg); CUMS + 20 mg/kg fluoxetine (fluoxetine). MOO (Beijing Tongrentang Company Limited, batch no. 2021B03527; 0.3 g MOO capsule containing 150 mg of bacitracin oligosaccharides) and fluoxetine (Aladdin, Cat. # F131623) were given by the gavage route, with a dosing volume of 0.1 mL/10 g once/day for 28 consecutive days. On day 29, a behavioral test was performed (Figure 1A). Subjects given to each group of mice were prepared using 0.9% saline. The dose of MOO used in this experiment was set according to the recommended amount for humans, and the dose of fluoxetine was referenced as previously described (Ma et al., 2024).

### 2.4 Behavioral assessments

#### 2.4.1 Sucrose preference test

Depressive symptoms were associated with a decrease in the percentage of sucrose preference test (SPT) in mice. The SPT procedure was carried out according to a previously described protocol by Su et al. (2024), with slight modifications. All mice were housed in single cages. Firstly, mice were given two bottles of 1% sucrose solution (w/v) of the same size and appearance for 24 h. Then, food and water were not provided for 16 h. Finally, food and two bottles of water (one bottle filled with 1% sucrose solution and the other with purified water) were provided, weighed, and randomly placed, and the positions were exchanged after 12 h. After 24 h, the two bottles of water were weighed, and the sucrose preference (SP) value was calculated using the following formula:


$$\begin{array}{l} \mathrm{Sucrose preference rate}\;\left(\%\right)\\ =\frac{\mathrm{Sucrose intake}\;\left(g\right)}{\left[\mathrm{Sucrose intake}\;\left(g\right)+\mathrm{Water intake}\;\left(g\right)\right]}\times 100\% \end{array}$$

#### 2.4.2 Tail suspension test

The tail suspension test (TST) procedure was carried out according to a previously described protocol by (Wang C. et al., 2024), with minor modifications. The experiments were performed in a soundproofed and visually isolated room. Each mouse was fixed with medical tape 1 cm distal to the tail on a stand so that its head was 50 cm above the floor for 6 min. The immobility time of each mouse was assessed during the last 5 min of the 6-min test period.

#### 2.4.3 Force swimming test

The force swimming test (FST) procedure was performed according to a previously described protocol by Jia et al. (2024), with minor modifications. The mice were placed in swimming buckets filled with water at 23 ± 1°C, 40 cm in height, 14 cm in diameter, and 30 cm in depth. One day before the start of the experiment, the mice were acclimatized by placing them in the bucket for 15 min, removing them from the water as soon as possible after swimming, drying them with a towel, and placing them in their respective cages. On the day of the experiment, the mice were placed in the bucket for 6 min, and the immobility time of the mice was recorded for the last 5 min.

#### 2.4.4 Open field test

The open field test (OFT) OFT process was carried out according to a previously described protocol by Zhou et al. (2024), with minor modifications. Mice were fixedly placed in the bottom of a black experimental box (42 × 42 × 42 cm; the bottom was evenly divided into 16 compartments); the central area of the open field was defined as the middle 4 compartments (20 × 20 cm); and the mice were allowed to move freely in the open field box for 6 min. The total distance the mice moved in the box and the total time they stayed in the central area were recorded for the last 5 min. Experimental procedures were recorded and analyzed using SMART 3.0 software.

#### 2.4.5 Sexual behavior test

The experimental procedure for sexual behavior was carried out according to a previously described protocol by Luan et al. (2024), with minor modifications. The experiment was conducted at night in a quiet, dimly lit environment. The experiment was conducted at night in a quiet, dimly lit environment. Male mice were placed individually in a clean cage, and after 5 min of acclimatization, female mice in estrus were placed in the cage. The parameters related to sexual behavior of male mice in each group were recorded for 30 min, including: mounting latency (ML), insertion latency (IL), mounting frequency (MF), insertion frequency (IF), and ejaculation latency (EL).

### 2.5 Hematoxylin eosin staining

Testicular and epididymal tissues from mice were collected immediately after behavioral testing and fixed in 4% paraformaldehyde for H$E staining. After dehydration, the tissues were embedded in paraffin, sectioned coronally using a pathology sectioning machine (Leica RM 2016, Leica Instruments Shanghai Ltd.) with a section thickness of approximately 4 μm, stained with HE, and sealed (Shen et al., 2024). The pathological features of each tissue of the mice were observed using panoramic section scanner software (Pannoramic desk/midi/250/1000, 3DHISTECH, Hungary).

### 2.6 Nissl staining

Nissl staining was performed as described previously by (Zhang Y. et al., 2024a), with minor adjustments. Each mouse was perfused intracardially with 0.9% saline, followed by 4% paraformaldehyde for 30 min. After fixing the brain, the mouse was embedded in paraffin and cut into 4 μm-thick sections by coronal sectioning. The sections were mounted on slides and baked in an oven at 65°C for 1 h. Sections were stained with 0.5% toluidine blue O for 10 min. All sections were imaged using a microscope (Nikon, Y-TV55). The number of Nissl bodies in the hippocampus was quantified using ImageJ software.

### 2.7 Enzyme linked immunosorbent assay

After behavior tests, the mice were sacrificed by cervical dislocation. Whole blood was left at room temperature for 2 h and then centrifuged at 1,000 g for 15 min, and the supernatant was collected. Hippocampal tissues were rapidly separated on ice, and the separated brain tissues were added to 1 mL of ice-cold 1× PBS solution to make a homogenate, which was placed at −20°C overnight. After 2 repeated freeze-thaw treatments to disrupt the cell membrane, it was centrifuged at 5,000 g for 5 min at 4°C, and the supernatant was collected for the experiment. Hippocampal 5-HT (CSB-E08365m), DA (CSB-E08661m), NE (CSB-E07870m) and 5-HIAA (E-EL-0075C) levels were detected using enzyme linked immunosorbent assay (ELISA) kits, purchased from CUSABIO (Wuhan, China), and serum T (E-OSEL-M0003), E_2_ (E OSEL-M0008), FSH (E-EL-M0511c) and LH (E-EL-M3053) levels, purchased from Elabscience (Wuhan, China), were performed according to the manufacturer’s instructions (Sheng et al., 2023). Optical density (OD) was measured at 450 nm using an enzyme marker to quantify the results, and the indicator content was determined using a standard curve.

### 2.8 Quantitative real-time PCR

Total RNA from hippocampal tissues was extracted using Trizol reagent according to the manufacturer’s method. The concentration and purity of extracted RNA were determined using nanodrop spectrophotometry for RNA quantification. Reverse transcription was performed to convert the total RNA into cDNA, and the expression level of the target gene was normalized to the mRNA level of GAPDH using SYBR Green qPCR Master Mix according to the manufacturer’s instructions. To ensure accurate normalization, the expression level of the target gene was normalized to the mRNA level of GAPDH (Zhang H. et al., 2024). Table 1 lists the primer sequences used in the present study.

**Table 1:** PCR sequence in this study.

Primers	Forward	Reverse
GAPDH	5′-CCTCGTCCCGTAGACAAAATG-3′	5′-TGAGGTCAATGAAGGGGTCGT-3′
BNDF	5′-GCCCATGAAAGAAGTAAACGTCC-3′	5′-AGTGTCAGCCAGTGATGTCGTC-3′
TrkB	5′-ATCACCAACAGTCAGCTCAAGC-3′	5′-TTCAGCGTCTTCACAGCCAC-3′
CREB	5′-TGGCTAACAATGGTACGGATGG-3′	5′-GTGCTGTGCGGATCTGGTATGT-3′

### 2.9 Western blot

Hippocampal tissue proteins were extracted using pre-cooled lysis buffer containing PMSF. Protein concentration was determined using a BCA protein kit (Dingguo Changsheng, China). For electrophoresis, hippocampal proteins were passed through a 10% polyacrylamide gel, and the proteins were separated by constant-pressure SDS-PAGE (40 μg of proteins were separated in each lane), and then the proteins were transferred to PVDF membranes and blocked with 5% BSA for 2 h. Primary antibodies included beta-actin antibody (Cell Signaling Technology; 4970; dilution ratio: 1:1,000), brain-derived neurotrophic factor (BDNF) antibody (Abcam; AB108319; dilution ratio: 1:10,000), tropomyosin receptor kinase B (TrkB) antibody (Abcam; ab187041; dilution ratio: 1:5,000), phosphorylated-TrkB Tyr705 antibody (Abcam; ab229908; dilution ratio: 1:1,000), cyclic adenosine monophosphate effector-binding protein (CREB) antibody (Cell Signaling Technology; 9197; dilution ratio: 1:1,000), and phosphorylated-CREB Ser133 antibody (Cell Signaling Technology; 9198; dilution ratio: 1:1,000). Samples were washed 3 times with TBST, incubated with anti-rabbit IgG (H + L) antibody (Beyotime; A0208; 1:1,000) for 2 h at room temperature, washed 3 times with TBST, and then left to develop (Zhao et al., 2024). The signal intensity of the protein bands was quantified using ImageJ.

### 2.10 16S rDNA amplicon sequencing

The method used for this experiment has been previously reported by Ouyang et al. (2024). At the end of behavioral studies, mouse feces were collected, and microbial DNA was extracted from the feces. The composition of the gut microbiota was analyzed by sequencing 16S r DNA amplicons. Primers 341F (CCTACGGGGNGGCWGCAG) and 806R (GGACTACHVGGGGTATCTAAT) were selected to amplify the V3–V4 region with an amplification length of 470 bp, to generate amplicons, and for taxonomic analysis. PCR amplicons were purified, identified, and bipartite sequenced, and sequences with greater than 97% similarity were obtained by clustering using QIIME 2 software in operable taxonomic units (OTUs). Each representative sequence within an OTU was classified, and the resulting data were sequentially spliced, filtered, clustered in OTUs, and computed to obtain information on the corresponding substances and their distribution.

### 2.11 Morphological analysis of spermatozoa in mice

It was performed as previously described by Fang et al. (2024), with minor adjustments. After the mice were sacrificed by cervical dislocation, the epididymis was put into saline, cut longitudinally along the tail, left to stand for 3–5 min, and then gently shaken, and the filtrate was taken and smeared. After drying, it was fixed with methanol for 5 min, stained with 1% eosin solution for 15–20 min, dried, and washed gently with water. Under the microscope, the part with less sperm overlapping and a clearer field of view was taken for morphological observation. For each mouse, 400 spermatozoa were observed, and the possible morphologies of spermatozoa included normal, abnormal heads, sperms with two heads, amorphous, folded tails, and double-tailed tails. The total number of malformed sperm was recorded, and the percentage of sperm malformation was calculated using the formula:


$$\begin{array}{l} \mathrm{Sperm abnormality rate}\\ =\frac{\mathrm{Total number of sperm abnormalities}}{\mathrm{Total number of sperm observed}}\times 100\% \end{array}$$

### 2.12 Statistical analysis

The data obtained were analyzed for significance using SPSS 19.0 statistical software. and the results were expressed in mean ± standard deviation (SD) form. Plotting was done using GraphPad Prism 9.5 software. Data are presented as mean ± standard deviation (SD) and analyzed using a paired *t*-test or one-way ANOVA, according to the different experiments. A *p*-value <0.05 indicated statistical significance.

## 3 Results

### 3.1 MOO improved depression-like behaviors in CUMS-induced mice

Behavioral experiments (SPT, FST, TST, and OFT), were performed to evaluate whether MOO had an antidepressant effect on mice. After 28 day of CUMS modeling and MOO treatment, mice in the CUMS group demonstrated a significant reduction in body weight when compared with controls (*p* < 0.01, Figure 1C), and less sugar-water preference (*p* < 0.01, Figure 1D), indicating a lack of pleasure, and a significant reduction in the total distance moved in the open field (*p* < 0.01, Figure 1G) and the residence time in the central area (*p* < 0.01, Figure 1H), indicating diminished spontaneous activity and exploratory behavior. In addition, CUMS significantly prolonged the mice’s immobility time in the TST (*p* < 0.01, Figure 1E) and FST (*p* < 0.01, Figure 1F), exhibiting decreased mobility and behavioral despair. Compared with the CUMS group, MOO-administered mice gained weight, especially those in the MOO-25 mg/kg group (*p <* 0.05), and exhibited a more active and healthy state. MOO significantly increased the degree of sucrose preference (*p <* 0.01), the total distance traveled in the open field (MOO-12.5 mg/kg, *p* < 0.05; MOO-25 mg/kg, *p* < 0.01), and residence time in the central area (*p* < 0.01), while shortening the immobility time of TST (MOO-12.5 mg/kg, *p* < 0.05; MOO-25 mg/kg, *p* < 0.01) and FST (*p* < 0.01), illustrating MOO’s potential to alleviate depression-like symptoms induced by CUMS in mice.

### 3.2 Neuroprotective effects of MOO on hippocampal neurons in CUMS-induced mice

**Figure 2:**
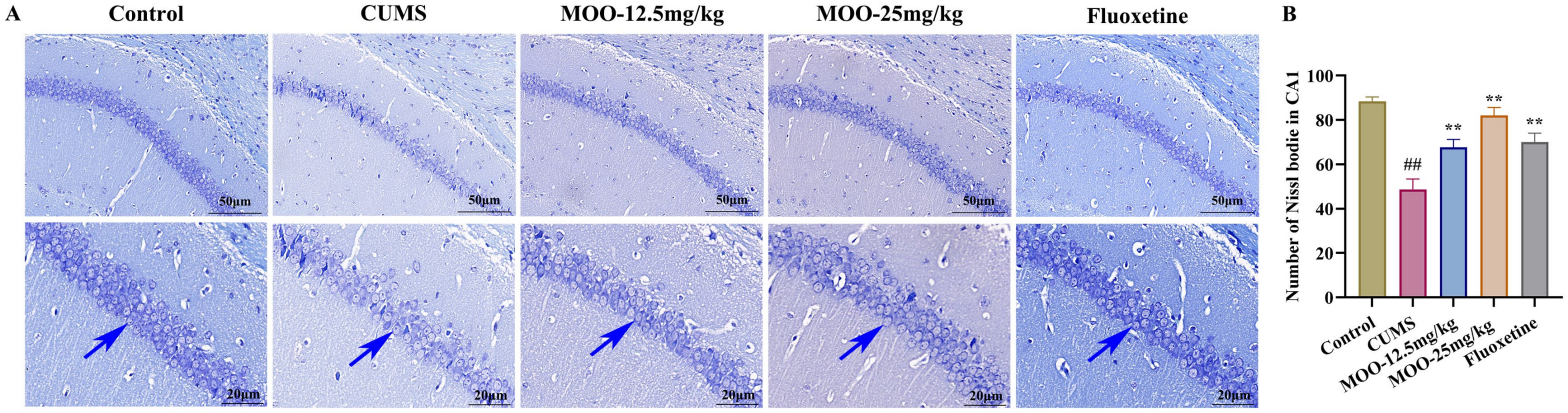
Neuroprotective effect of MOO on hippocampal neurons in CUMS-induced mice. **(A)** Nissl-stained neurons in the hippocampal CA1 region, scale bar: 50 μm and 20 μm; **(B)** Number of Nissl bodies in the hippocampal CA1 region. Blue arrows indicate changes in Nissl bodies in the CA1 region of the hippocampus. Results are expressed as mean ± SD (*n* = 3). ^##^*p* < 0.01 vs. control group; ^**^p < 0.01 vs. CUMS group.

Nissl bodies, a characteristic structure of the nucleus accumbens, are considered one of the main indicators of hippocampal neuronal damage and are closely associated with impaired neuroplasticity (Chang et al., 2024). The number of Nissl bodies is also strongly associated with the development of depression. When neurons are stimulated, Nissl bodies in the cells are significantly reduced or even lost. Therefore, the presence and disappearance of Nissl bodies can reflect whether neurons are damaged or not. Studies have pointed out that neuronal damage in the CA1 region of the hippocampus leads to learning and memory deficits (Liu et al., 2023). Interestingly, Nissl bodies disintegrated, and the number of Nissl bodies was significantly reduced in the hippocampal CA1 region in the CUMS group compared to the control group (*p* < 0.01, Figure 2). MOO treatment significantly increased the number of Nissl bodies in the hippocampal CA1 region of CUMS-induced mice compared to the CUMS group (MOO-12.5 mg/kg, *p* < 0.01; MOO-H, *p* < 0.01). The results indicated that MOO treatment attenuated CUMS-induced neuronal damage in the mice hippocampus and contributed to the survival of hippocampal neuronal cells in depressed mice.

### 3.3 Effect of MOO on monoamine neurotransmitters in CUMS-induced mice

**Figure 3:**
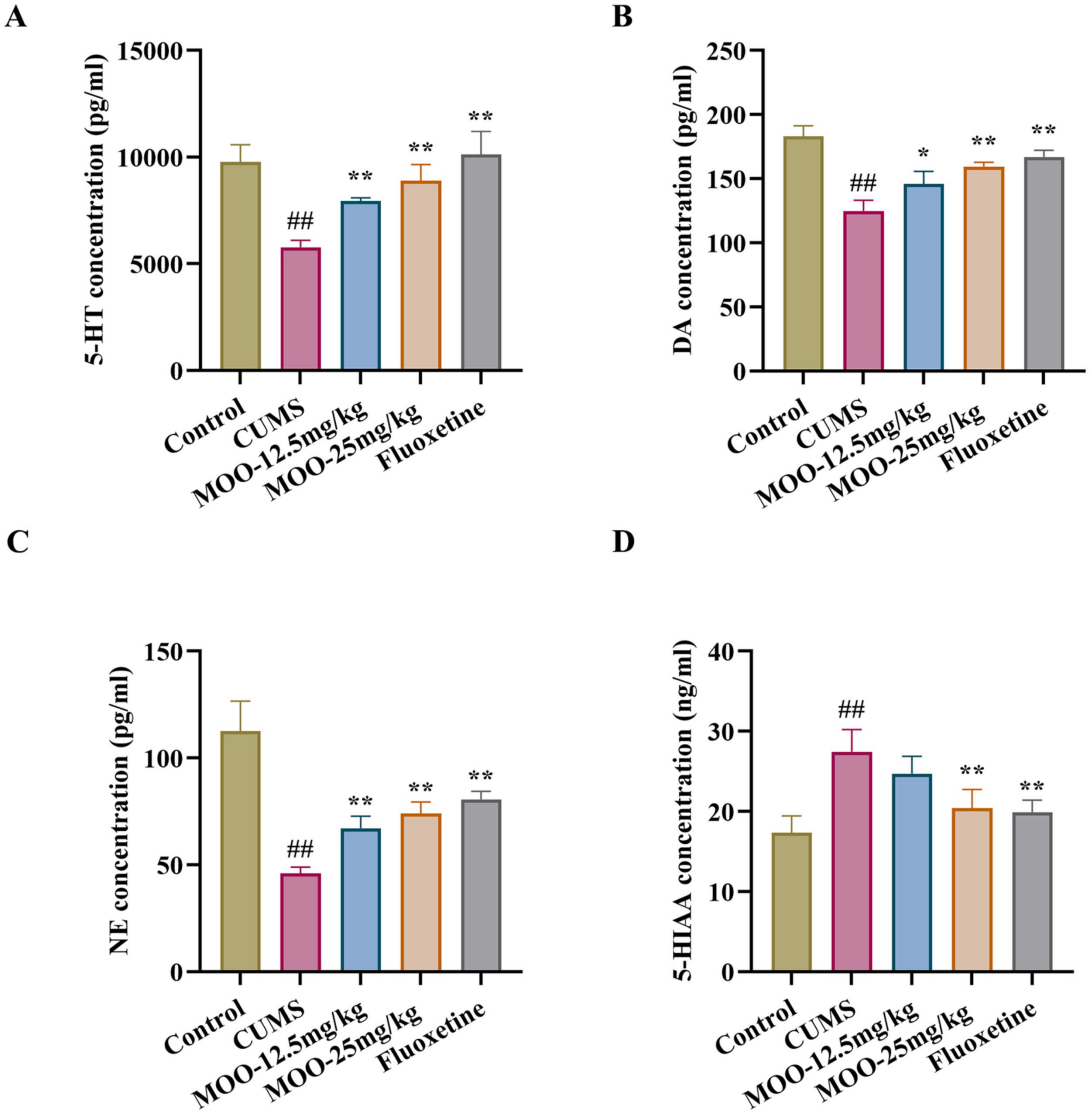
Effect of MOO on monoamine neurotransmission in CUMS-induced mice. **(A–D)** ELISA detection of the expression levels of monoamine neurotransmitters 5-HT **(A)**, NE **(B)**, DA **(C)**, and 5-HIAA **(D)** in hippocampal tissue. Results are expressed as mean ± SD (*n* = 3). ^##^*p* < 0.01 vs. control group; ^*^p < 0.05; and ^**^p < 0.01 vs. CUMS group.

Neurotransmitters such as serotonin are biomolecules that transmit signals between neurons and target cells. They regulate the process of various physiological and psychological activities, which are closely related to the pathogenesis of depression. Compared with the control group, the levels of 5-HT (*p* < 0.01, Figure 3A), NE (*p* < 0.01, Figure 3B), and DA (*p* < 0.01, Figure 3C) in the CUMS group were significantly lower, and the 5-HIAA content was significantly higher (*p* < 0.01, Figure 3D) in the hippocampus. However, after 4 weeks of MOO treatment, the levels of 5-HT (MOO-12.5 mg/kg, *p* < 0.01; MOO-25 mg/kg, *p* < 0.01), NE (MOO-12.5 mg/kg, *p* < 0.01; MOO-25 mg/kg, *p* < 0.01), and DA (MOO-12.5 mg/kg, *p* < 0.05; MOO-25 mg/kg, *p* < 0.01) were significantly elevated, while the 5-HIAA levels were significantly decreased (MOO-12.5 mg/kg, *p* > 0.05; MOO-25 mg/kg, *p* < 0.01), when compared with CUMS mice, suggesting that MOO restores dysregulation of monoamine neurotransmitter levels in the hippocampus.

### 3.4 Effect of MOO on the BDNF/TrkB/CREB pathway in the hippocampus of CUMS-induced mice

**Figure 4:**
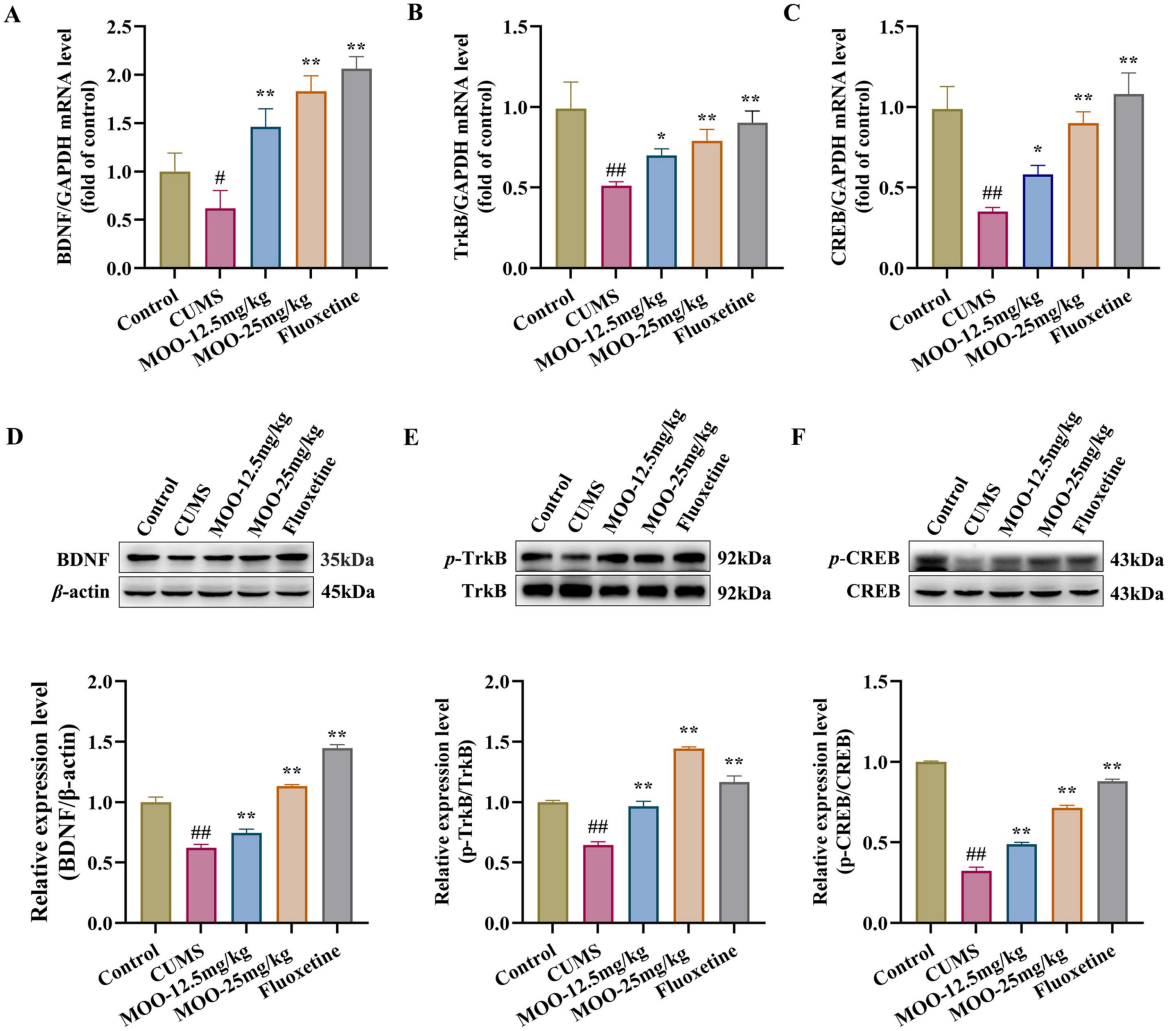
Effect of MOO on the BDNF/TrkB/CREB signaling pathway in the hippocampus of mice with CUMS-induced depression. **(A–C)** RT-qPCR analysis of BDNF **(A)**, TrkB **(B)**, and CREB **(C)** expression in the hippocampus. **(D–F)** Western blot analysis of BDNF **(D)**, *p*-TrkB **(E)**, and *p*-CREB **(F)** expression in the hippocampus. Data are expressed as mean ± SD (*n* = 3). ^##^*p* < 0.01 vs. control group; ^*^p < 0.05; and ^**^p < 0.01 vs. CUMS group.

BDNF promotes the growth and development of immature neurons, enhances the survival and function of adult neurons, contributes to the maintenance of synaptic connectivity, and is a key regulator of neurogenesis and synaptic plasticity (Begni et al., 2017). Alterations in BDNF in the brain often act as an indicator of social stress on the brain physiology and systematic effects (Han et al., 2025). RT-qPCR analysis was performed to examine the mRNA expression levels of the proteins associated with the BDNF/TrkB/CREB signaling pathway in the hippocampus of mice in each group. As shown in Figures 4A–C, compared to the control group, the mRNA expression levels of BDNF (*p* < 0.05, Figure 4A), TrkB (*p* < 0.01, Figure 4B), and CREB (*p* < 0.01, Figure 4C) proteins were significantly reduced in the hippocampus of the CUMS group. Similarly, BDNF (MOO-12.5 mg/kg, *p* < 0.01; MOO-25 mg/kg, *p* < 0.01), TrkB (MOO-12.5 mg/kg, *p* < 0.05; MOO-25 mg/kg, *p* < 0.01), and CREB (MOO-12.5 mg/kg, *p* < 0.05; MOO-25 mg/kg, *p* < 0.01) protein mRNA expression levels were significantly higher in the hippocampus after the MOO administration.

Western blot analysis was performed to detect the expression of proteins associated with the BDNF/TrkB/CREB pathway in the hippocampus of mice in each group. *p*-TrkB and *p*-CREB protein levels were normalized to *β*-actin and total TrkB and CREB protein levels, respectively. As shown in Figures 4D–F, BDNF (*p* < 0.01, Figure 4D), *p*-TrkB/TrkB (*p* < 0.01, Figure 4E), and *p*-CREB/CREB (*p* < 0.01, Figure 4F) expression were significantly reduced in the hippocampus of the CUMS group compared to the control group. Similarly, BDNF (MOO-12.5 mg/kg, *p* < 0.01; MOO-25 mg/kg, *p* < 0.01), *p*-TrkB/TrkB (MOO-12.5 mg/kg, *p* < 0.01; MOO-25 mg/kg, *p* < 0.01), and *p*-CREB/CREB (MOO-12.5 mg/kg, *p* < 0.01; MOO-25 mg/kg, *p* < 0.01) expressions were significantly higher in the hippocampus after MOO treatment compared to CUMS.

### 3.5 MOO had a regulatory effect on the gut microbiota in CUMS-induced mice

#### 3.5.1 Effect of MOO on the diversity of gut microbiota in CUMS-induced mice

**Figure 5:**
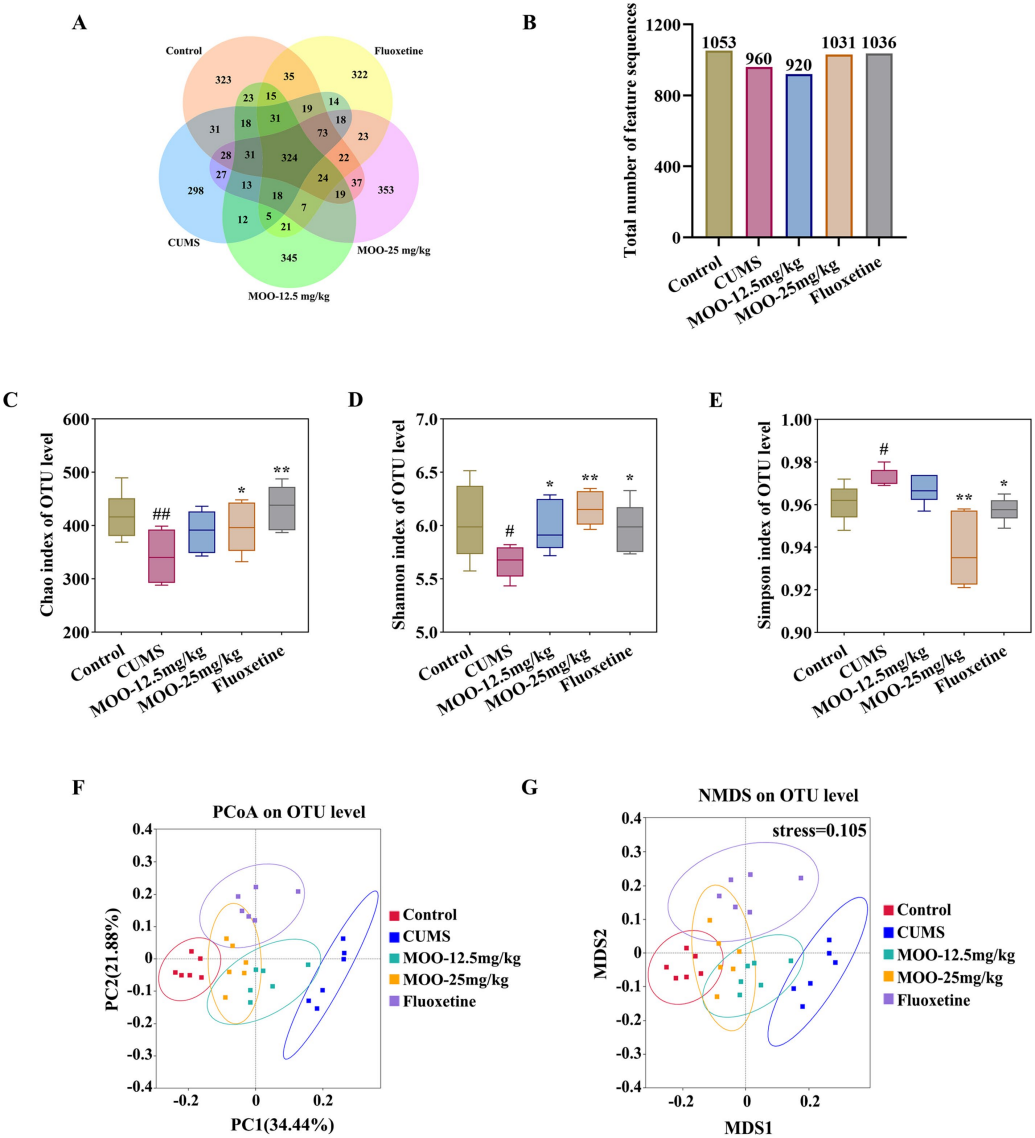
Effect of MOO on the diversity of gut microbiota in CUMS-induced mice. **(A,B)** Wayne’s diagram of OTU core discs and their quantitative values. **(C–E)** Effect of MOO on α-diversity of gut microbiota in mice, Chao’s index **(C)**, Shannon’s index **(D)**, and Simpson’s index **(E)**. **(F,G)** Effect of MOO on β-diversity of gut microbiota in mice. Results are expressed as mean ± SD (*n* = 6). ^#^*p* < 0.05, ^##^*p* < 0.01, vs. control group; ^*^p < 0.05, ^**^p < 0.01 vs. CUMS group.

In order to investigate the possible involvement of gut microbiota in the antidepressant mechanism of MOO, fecal samples from mice were analyzed by 16S rRNA sequencing. A comparison of gut microbial OTUs was performed based on the obtained feature sequence results. The results are shown to obtain the number of OTUs for the five groups of samples in the present study for which petal OTU core disk mapping was performed at 97% similarity (Figures 5A,B).

**Figure 6:**
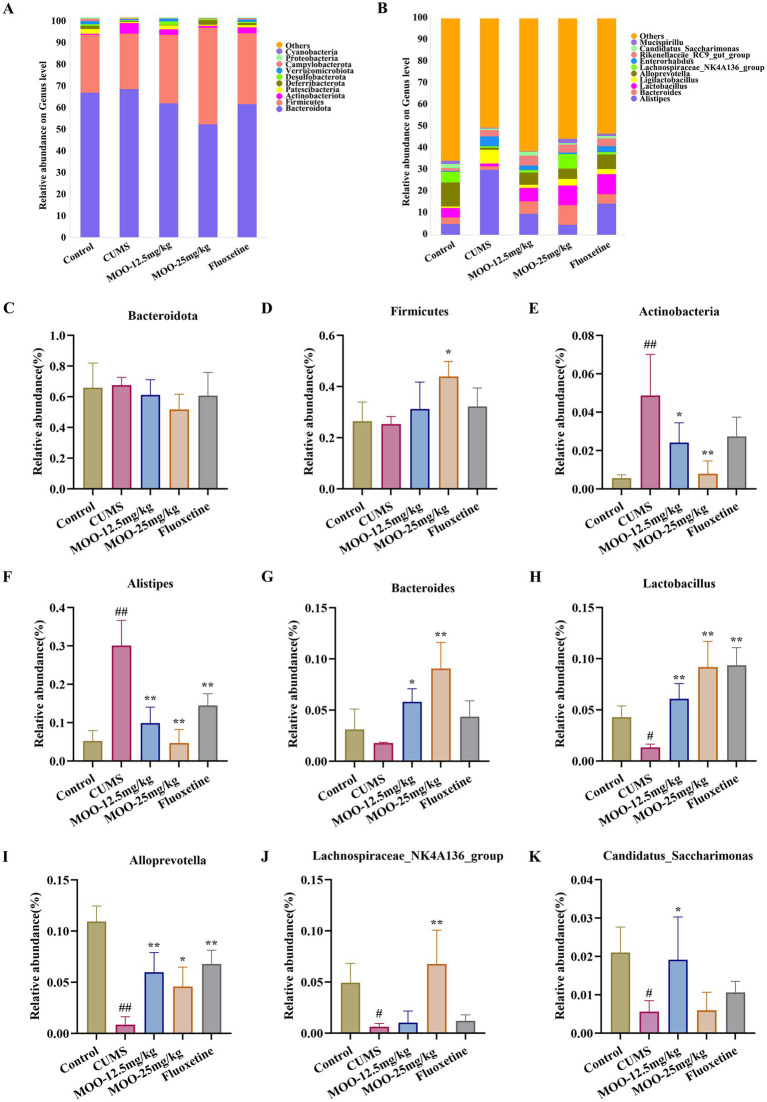
Effect of MOO on the composition of gut microbiota in CUMS-induced mice. **(A)** Image of abundance at the phylum level. **(C–E)** Species abundance at the phylum level. **(B)** Image of abundance at the genus level. **(F–K)** Species abundance at the genus level. Results are expressed as mean ± SD (*n* = 6). ^#^*p* < 0.05, ^##^*p* < 0.01 vs. control group; ^*^p < 0.05, ^**^p < 0.01 vs. CUMS group.

Differential species were analyzed for each group. The phylum-level analysis showed that the gut microbiota consisted of four major phyla, namely Bacteroidota, Firmicutes, Actinobacteria, and Proteobacteria, which accounted for more than 96% in all groups (Figure 6A). It was shown that the abundance of Actinobacteria and Proteobacteria could reflect the balance of the gut microbiota. The genus level analysis showed that the 10 dominant genera were Alistipes, Bacteroides, Lactobacillus, Ligilactobacillus, Alloprevotella, Lachnospiraceae_NK4A136_group, Enterorhabdus, Rikenellaceae_RC9_gut_group, and Candidatus_Saccharimonas Mucispirillum (Figure 6B).

The *α*-diversity of the mouse gut microbiota was further analyzed. Compared with the control group, the Chao index (*p* < 0.01, Figure 5C) and Shannon index (*p* < 0.05, Figure 5D) were significantly lower and the Simpson index was significantly higher (*p* < 0.05, Figure 5E) in the CUMS group, which indicated that the abundance and diversity of the gut microbiota of the CUMS mice were reduced. Compared with the CUMS group, the Chao index (MOO-12.5 mg/kg, *p* > 0.05; MOO-25 mg/kg, *p* < 0.05) and Shannon index (MOO-12.5 mg/kg, *p* < 0.05; MOO-25 mg/kg, *p* < 0.01) were significantly higher, and the Simpson index (MOO-12.5 mg/kg, *p* > 0.05; MOO-25 mg/kg, *p* < 0.01) was significantly lower in the MOO group, suggesting that MOO significantly improved the structure of the gut microbiota in the CUMS-depressed mice and indicating an increase in its diversity and richness.

The β-diversity analysis was used to measure differences in microbial community composition between samples. The results for PCoA (based on weighted UniFrac distance) and NMDS (based on weighted UniFrac distance), as shown in Figure 5, illustrated that the microbiota was distributed in different areas between the control group and the CUMS group, suggesting that the control group and the CUMS group had significantly segregated clusters. After intervention by MOO, the microbiota distribution of MOO mice was similar to that of the control group, suggesting that MOO improves the gut microbiota composition in CUMS-induced depressed mice.

#### 3.5.2 Effect of MOO on the composition of gut microbiota in CUMS-induced mice

CUMS alters the composition and abundance of the gut microbiota, resulting in a decrease in beneficial bacteria and an increase in harmful bacteria. Differential species were analyzed in each group. The phylum-level analysis showed a decrease in the abundance of Firmicutes (*p* > 0.05, Figure 6D) and an increase in the abundance of Bacteroidota (*p* > 0.05, Figure 6C) and Actinobacteria (*p* < 0.01, Figure 6E) in the CUMS group of mice compared with the control group of mice. Administration of MOO reversed the changes in the relative abundance of Bacteroidota (MOO-12.5 mg/kg, *p* > 0.05; MOO-25 mg/kg, *p* > 0.05), Firmicutes (MOO-12.5 mg/kg, *p* > 0.05; MOO-25 mg/kg, *p* < 0.05), and Actinobacteria (MOO-12.5 mg/kg, *p* < 0.05; MOO-25 mg/kg, *p* < 0.01), which indicated that MOO had a significant effect on the gut disorders in mice and had some ameliorating effect.

The analysis of genus levels showed that CUMS stimulation resulted in disturbed gut microbiota with increased levels of Alistipes (*p* < 0.01, Figure 6F) in the gut compared to the control group. Bacteroides (*p* > 0.05, Figure 6G), Lactobacillus (*p* < 0.05, Figure 6H), Alloprevotella (*p* < 0.01, Figure 6I), Lachnospiraceae_NK4A136_group (*p* < 0.05, Figure 6J), and Candidatus_Saccharimonas (*p* < 0.05, Figure 6K) Mucispirillum were reduced in the levels of the genera. MOO partially restored gut microbiota disorders by affecting the composition of the gut microbiota, resulting in a decrease in the relative abundance of Alistipes (MOO-12.5 mg/kg, *p* < 0.01; MOO-25 mg/kg, *p* < 0.01) in the gut tract compared to the CUMS group, and the relative abundance of Bacteroides (MOO-12.5 mg/kg, *p* < 0.05; MOO-25 mg/kg, *p* < 0.01), Lactobacillus (MOO-12.5 mg/kg, *p* < 0.01; MOO-25 mg/kg, *p* < 0.01), Alloprevotella (MOO-12.5 mg/kg, *p* < 0.01; MOO-25 mg/kg, *p* < 0.05), Lachnospiraceae_NK4A136_group (MOO-12.5 mg/kg, *p* > 0.05; MOO-25 mg/kg, *p* < 0.01), and Candidatus_Saccharimonas (MOO-12.5 mg/kg, *p* < 0.05; MOO-25 mg/kg, *p* > 0.05) Mucispirillum increased levels of the genera.

The above results suggest that the antidepressant effect of MOO observed in CUMS mice may be due to changes in the composition of the gut microbiota, whose abundance changed after MOO treatment, especially the reduction in the abundance of bacteria associated with depression, which alleviated depressive symptoms.

### 3.6 Effect of MOO on sexual and behavioral competence in CUMS-induced mice

As shown in Table 2, compared with the control group, mice in the CUMS group demonstrated a significant decrease in the number of mounts (*p* < 0.01) and a significant extension of mount latency (*p* < 0.01), indicating that the mating desire of depressed mice was significantly reduced, and mice in the CUMS group showed a significant decrease in the number of insertions (*p* < 0.01) and a significant prolongation of insertion latency (*p* < 0.01) and ejaculation latency (*p* < 0.01), suggesting that the depression sexual performance was also significantly reduced. Surprisingly, all the observed indexes improved after MOO treatment, while all indices in the MOO-25 mg/kg group were significant (*p* < 0.01) and close to those of the control group, suggesting that MOO could improve the libido and enhance the sexual ability of depressed mice.

**Table 2:** Effect of MOO on sexual behavioral competence in CUMS-induced mice.

Group	Mounting frequency	Mounting latency (s)	Insertions frequency	Insertion latency (s)	Ejaculation latency (s)
Control	7.00 ± 1.33	165.19 ± 56.55	6.30 ± 1.16	377.51 ± 100.01	538.03 ± 128.92
CUMS	4.40 ± 1.65^##^	368.48 ± 78.56^##^	4.00 ± 1.49^##^	650.58 ± 138.02^##^	1066.11 ± 180.74^##^
MOO-12.5 mg/kg	5.70 ± 1.34	308.00 ± 54.55	5.10 ± 1.37	552.62 ± 143.36	758.10 ± 201.68^**^
MOO-25 mg/kg	7.70 ± 1.34^**^	200.20 ± 80.12^**^	6.40 ± 1.27^**^	410.53 ± 107.11^**^	583.69 ± 172.51^**^
Fluoxetine	6.20 ± 1.55^**^	249.14 ± 74.95^**^	5.8 ± 1.40^**^	460.87 ± 127.03^**^	627.65 ± 172.51^**^

Results are expressed as mean ± SD; ^##^*p* < 0.01 vs. control group; ^**^p < 0.01 vs. CUMS group (*n* = 10).

### 3.7 Effect of MOO on histopathological changed in the sex organs of CUMS-induced mice

**Figure 7:**
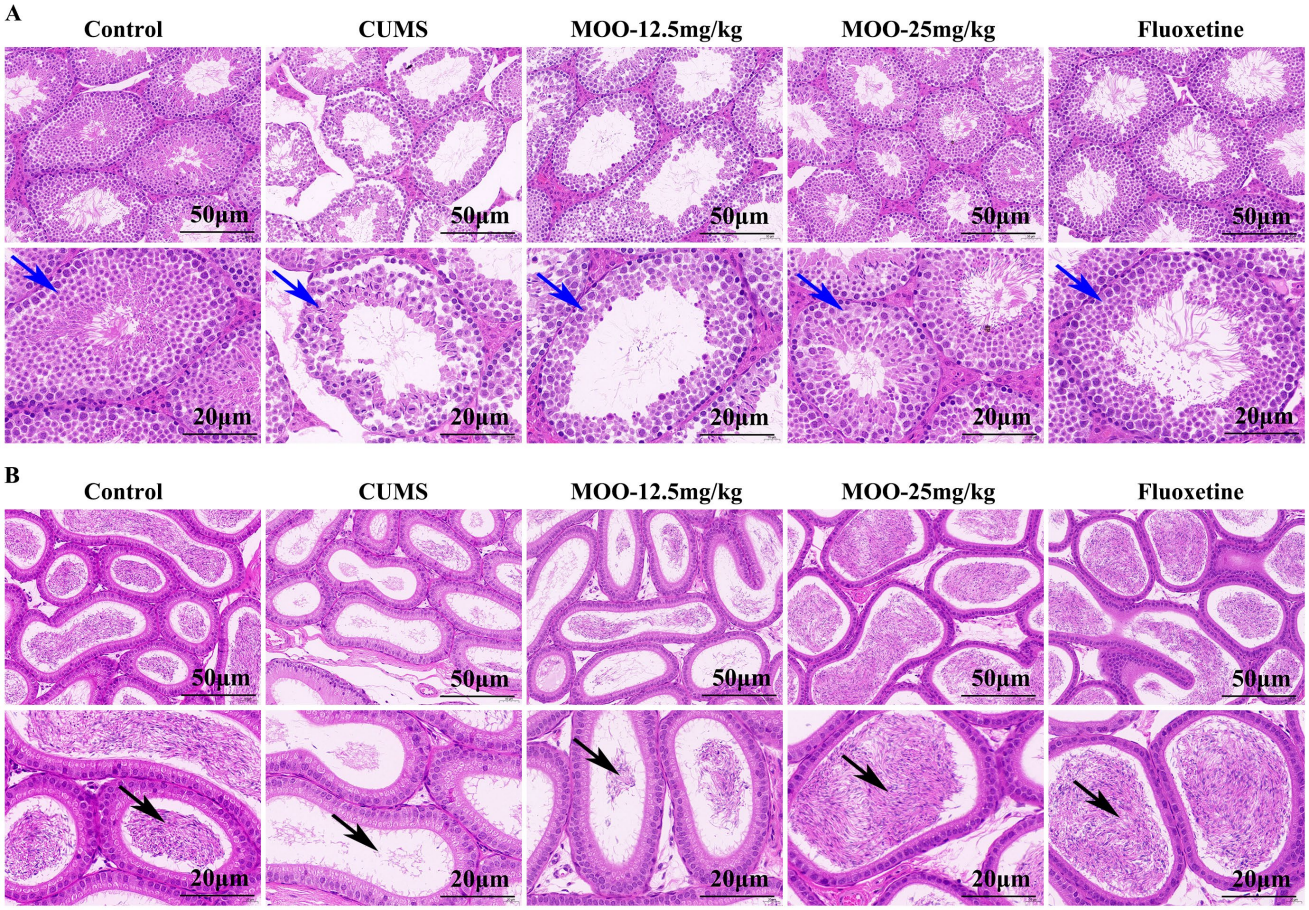
Effect of MOO on histopathological changed in the sex organs of CUMS-induced depressed mice. **(A)** H&E staining evaluation of testis tissue, scale bar: 50 μm, and 20 μm. **(B)** H&E staining evaluation of epididymis tissue, scale bar: 50 μm, and 20 μm (*n* = 3). Blue arrows indicate the number of layers and arrangement of spermatogenic cells within the seminiferous tubules of testicular tissue; black arrows indicate the lumen of epididymal tissue containing spermatozoa and secretions.

Hematoxylin eosin (H&E) staining of the testicular tissue of CUMS-induced depressed mice is shown (Figure 7A). The testicular tissues of control mice had abundant spermatogenic tubules with regular arrangement, complete spermatogenic epithelium in the spermatogenic tubules, abundant spermatogenic cells, and normal morphology for supporting cells in the spermatogenic tubules. Spermatogonia and spermatozoa were visible in the lumen of the tubules. Compared with the control group, the testicular tissues of mice in the CUMS group showed severe vacuolization of the spermatogenic tubules, disordered arrangement of spermatogenic cells in the tubules, and obvious shedding of spermatogenic cells. After treatment with different concentrations of MOO, the above conditions were significantly improved. Compared to the CUMS group, after 25 mg/kg of MOO treatment, the number of spermatogenic cells was sufficient, the shape was regular, the spermatogonia and spermatozoa in the lumen increased significantly, and the vacuolization in the lumen of the spermatid tubules disappeared significantly. Thus, the results indicate that MOO was able to ameliorate the pathological damage produced by CUMS-induced depression in the testis of mice.

H&E staining of epididymal tissue of CUMS-induced depressed mice is shown (Figure 7B). The pseudo complex columnar epithelium of the epididymal ducts in the control mice was intact, with tightly arranged cells, normal morphology, and the lumen containing spermatozoa and secretion. Compared to the control group, the lumen of epididymal tissue in mice in the CUMS group contained very few spermatozoa and secretions, and some tissues were loosely arranged. These conditions were significantly improved after treatment with different concentrations of MOO. Among them, the lumen of epididymal tissue contained significantly more spermatozoa and secretions, and the cells were tightly arranged after treatment with 25 mg/kg of MOO compared with that of the CUMS group, revealing that MOO was able to improve the ability of epididymal testis to store spermatozoa in CUMS-induced depressed mice.

### 3.8 Effect of MOO on sex hormone secretion and sperm morphology in CUMS-induced depressed mice

**Figure 8:**
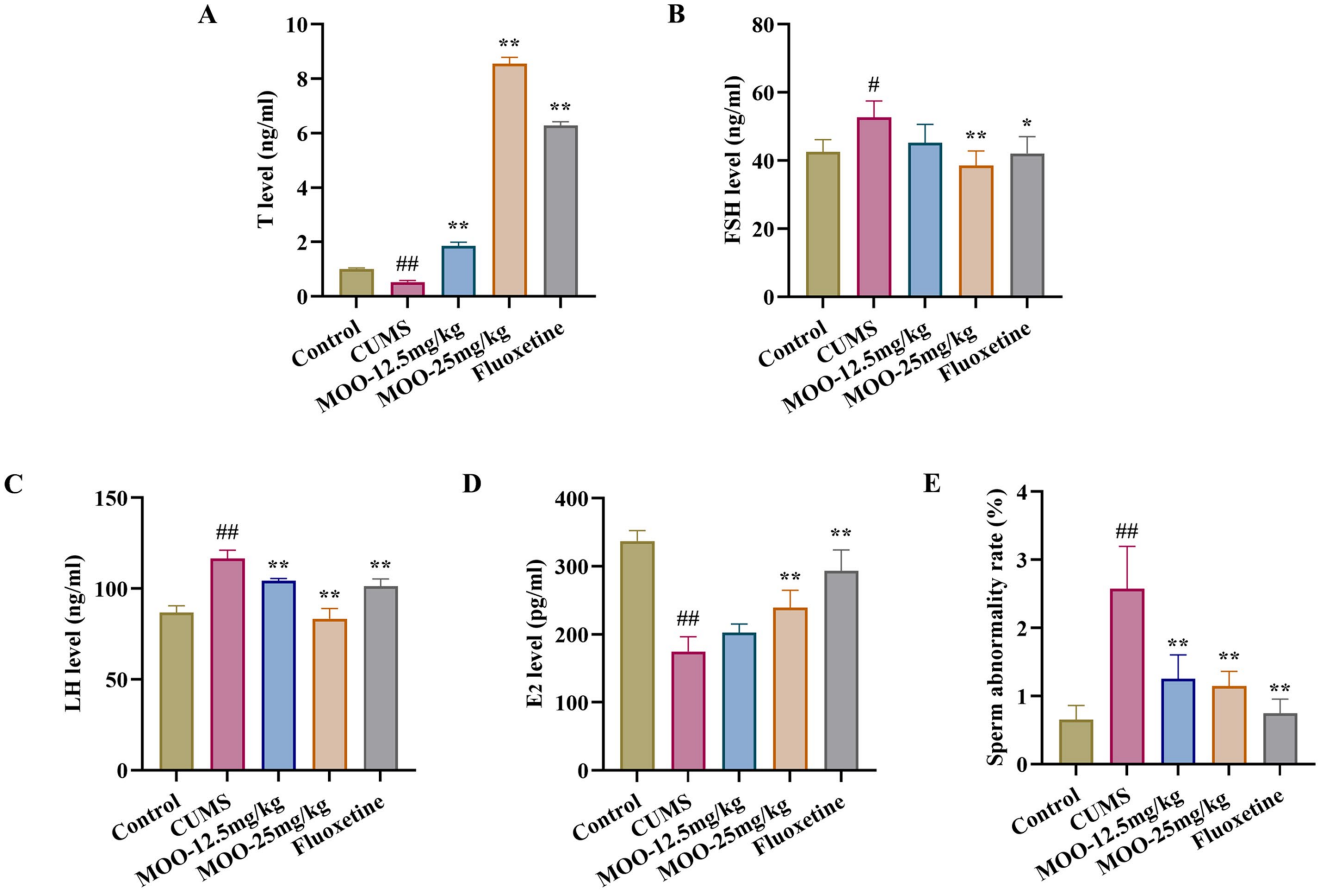
Effect of MOO on sex hormone secretion and sperm morphology in CUMS-induced depressed mice. **(A–D)** ELISA assay detection of the expression levels of sex hormone T **(A)**, FSH **(B)**, LH **(C)**, and E_2_ **(D)** in mice serum. **(E)** Sperm deformities in the epididymis. Results are expressed as mean ± SD (*n* = 3). ^#^*p* < 0.05, ^##^*p* < 0.01 vs. control group; ^*^p < 0.05, ^**^p < 0.01 vs. CUMS group.

In animal models of sexual dysfunction, serum levels of T, LH, FSH, and E_2_ are also important indicators for assessing the strength of sexual ability. Disorders of sex hormone levels may also lead to hypogonadism (Huang et al., 2023). As shown in Figure 8, the serum levels of T (*p* < 0.01, Figure 8A) and E_2_ (*p* < 0.01, Figure 8D) were lower in the CUMS group of mice than in the control group, and the levels of FSH (*p* < 0.05, Figure 8B) and LH (*p* < 0.01, Figure 8C) were higher than in the control group. Compared with CUMS, serum T (MOO-12.5 mg/kg, *p* < 0.01; MOO-25 mg/kg, *p* < 0.01) and E_2_ (MOO-12.5 mg/kg, *p* > 0.05; MOO-25 mg/kg, *p* < 0.01) levels were significantly higher, and FSH (MOO-12.5 mg/kg, *p* > 0.05; MOO-25 mg/kg, *p* < 0.01) and LH (MOO-12.5 mg/kg, *p* < 0.01; MOO-25 mg/kg, *p* < 0.01) levels were restored to near-normal levels in the MOO group, suggesting that MOO has the effect of restoring CUMS-stimulation-induced gonadotropic function in depressed mice and the stability of hormone levels.

In addition, evaluation of the effect of MOO on sperm malformations in mice by observing morphological changes in spermatozoa. Sperm malformation rate was significantly higher in CUMS mice compared to controls (*p* < 0.01, Figure 8E). However, a decrease in the number of sperm malformations in the mice could be significantly observed under the intervention of MOO (MOO-12.5 mg/kg, *p* < 0.01; MOO-25 mg/kg, *p* < 0.01), suggesting that MOO was able to ameliorate sperm malformations in the epididymis of CUMS-induced depressed mice.

## 4 Discussion

Chronic and low-intensity stressor stimuli from external sources are one of the important causing factors of depression. The CUMS animal model simulates a variety of stressful stimuli faced by people in today’s society and has been widely used in depression research (Wang P. et al., 2024). As a common clinical auxiliary therapy, TCM has been reported to have a therapeutic role in the regulation of emotions and sexual activity. *Morinda officinalis* is the main ingredient in TCM prescriptions for the treatment of depression and sexual dysfunction. As the main active ingredient of *Morinda officinalis*, MOO has been reported to have antidepressant effects in mice. However, it remains to be explored whether this effect involves modulation of sexual activity.

Previous studies have shown that prolonged exposure to social stress may reduce central parasympathetic neurotransmitters and also cause mating disorders and depression secondary to neurovascular lesions (Wu et al., 2023). There is growing evidence that monoamine transmitters (NE, 5-HT, and DA) are disturbed and are at reduced levels in the brains of depressed animals (Yu et al., 2024). Zhang et al. (Zhang Z. et al., 2024) revealed for the first time that MOOs administered orally to SD rats could act on intestinal flora and then pass the blood-brain barrier to promote the production of 5-HT in the brain and exert antidepressant effects. In addition, some animal experiments have shown that atractylenolide I (ATR) can act as an inhibitor by binding to 5-HT2A, and the knockdown of 5-HT2A ATR failed to further improve CUMS behaviors. ATR improved the behaviors of CUMS-induced depression-like phenotype mice by targeting 5-HT2A, thereby increasing the levels of neurotransmitters 5-HT, DA, and NE (Pei et al., 2024). Consistent with the effects reported in previous studies, our results indicate that imbalances in hippocampal 5-HT, DA, and NE levels were restored by MOO treatment in male mice experiencing chronic stress, thereby alleviating CUMS-induced depression-like behaviors.

Reduced BDNF is another important factor in the pathogenesis of depression (Zou et al., 2024). Studies have shown that BDNF and its receptor, tropomyosin receptor kinase B (TrkB), play a key role in the pathology of depression and the mechanism of antidepressant action (He et al., 2023). CREB (cyclic adenosine monophosphate effector binding protein), as a transcription factor, can form a CREB and BDNF/TrkB positive feedback loop with BDNF and TrkB in the nucleus to regulate a variety of functions such as cell survival, synaptic structure, and synaptic plasticity (Lu et al., 2022). Studies have shown that hydroxytyrosol activation of the BDNF/TrkB signaling pathway significantly ameliorates mitochondrial ultrastructural damage. Meanwhile, hydroxytyrosol also promotes nerve fiber function, myelin formation, microglia differentiation, and nerve regeneration through the BDNF/TrkB signaling pathway (Li et al., 2024). Notably, it has been demonstrated that 5-HT and BDNF act synergistically in regulating synaptic plasticity, with 5-HT triggering the expression of BDNF and BDNF improving the growth and survival of 5-HT neurons and maintaining synaptic plasticity in the adult brain (Hong et al., 2023). Our results showed that MOO increased BDNF expression in the hippocampus by mediating the BDNF/TrkB/CREB pathway and, at the same time, increased the number of hippocampal Nissl bodies, which contributed to the survival of hippocampal neuronal cells. These studies elucidate the molecular mechanisms underlying the neuroprotection of MOO in the treatment of depression.

In recent years, a growing body of research has supported the idea that imbalances in the microbiota-gut-brain dysfunction may contribute to the development of depression, and the gut microbiota may influence the development of depression through the brain-gut-microbiota axis (Wang J. et al., 2024). The gut microbiota has been reported to be able to influence the host psyche through the production of characteristic metabolites, which may affect basic neurodevelopmental processes at the blood-brain barrier, myelin sheaths, neurulation, and microglial cell maturation, with impacts on neurological development and function, as well as the ability to influence host behavior (Zhang et al., 2022). Studies have shown a link between gut microbiota dysbiosis and cognitive dysfunction, highlighting the specific taxa (Bacteroides and Alistipes) as potential factors for cognitive enhancement (Frileux et al., 2024). Relevant studies have shown that Alloprevotella rava, a bacterium known to produce compounds that improve brain health, is at lower levels in organisms with recent suicidal thoughts (Ahrens et al., 2022). Maternal glyphosate exposure has been reported to significantly alter the abundance of gut microbiota in female offspring, resulting in an increase in the abundance of the bacteria Alistipes and Blautia, which are involved in tryptophan metabolism and have been associated with depression and anxiety-like disorders in the gut (Buchenauer et al., 2023). A related study showed that dietary supplementation with *Macleaya cordata* extract (MCE) significantly increased the abundance of short-chain fatty acid-producing bacteria (Lachnospiraceae_NK4A136_group) in the cecum (Song et al., 2023). Notably, a specific family of bacteria, including Alloprevotella, showed a strong symbiotic relationship with brain tryptophan metabolomics, suggesting a potential mediating role of gut microbiome alterations and metabolites in the efficacy of XTN therapy (Wang X. et al., 2024). In our study, MOO treatment significantly reduced Alistipes and increased Alloprevotella and Lachnospiraceae_NK4A136_group in depressed mice. In addition, Zhang et al. (Zhang et al., 2022) study revealed for the first time that MOO positively regulates the tryptophan (Trp) → 5-hydroxytryptophan (5-HTP) → 5-hydroxytryptophan (5-HT) metabolic pathway through gut microbiota. Among them, peripheral 5-HT could not pass the blood-brain barrier (BBB), while 5-HTP could penetrate the BBB efficiently, and its study showed that MOO inhibited the activity of 5-hydroxytryptophan acid decarboxylase (5-HTPDC) by increasing the level of tryptophan hydroxylase (TPH) in the gut tissues to promote the metabolism of Trp for the generation of 5-HTP and, at the same time, inhibited the conversion of 5-HTP to 5-HT, which also inhibits the conversion of 5-HTP to 5-HT, resulting in the accumulation of 5-HTP in the gut tract. 5-HTP is absorbed into the bloodstream through the intestines and then enters the brain through the BBB to be rapidly metabolized to 5-HT, so that the content of 5-HT in the brain is increased and thus exerts an antidepressant effect. These studies have elucidated that MOO regulates the microbiota-gut-brain axis, thereby playing a role in the pathogenesis of depression, and is now an important target for preventing and slowing the development of depression.

Sexual dysfunction, such as decompensation or erectile dysfunction, is common in depressed patients, is present in approximately 20–50% of untreated depressed men, and is associated with the severity, duration, and recurrence of depressive episodes (Fischer et al., 2019). In stressful situations, the function of the hypothalamic–pituitary-gonadal (HPG) axis may be disturbed, leading to alterations in serum follicle-stimulating hormone (FSH) and luteinizing hormone (LH), leaving associated testosterone levels deficient (Xu et al., 2016). Studies have shown that gonadal steroids affect sexual function and that hormonal deviations in the HPG axis may be responsible for hypogonadism and erectile dysfunction in some depressed patients (EImazoudy et al., 2020). In addition, animal studies have shown that chronic stress impairs spermatogenesis and epididymal tissue in mice and induces decreased sexual performance, testicular damage, androgen levels, and semen quality parameters in male mice (Choowong-In et al., 2022). Relevant studies have shown that oligosaccharides isolated from the roots of *Morinda officinalis* F. C. can enhance the sexual function of healthy male mice, improve the mating ability and fertility of mice, increase the level of testosterone in the testes, and play a protective and therapeutic role in male mice with reproductive dysfunction, in agreement with our findings (Wu et al., 2015). Our results showed that MOO treatment improved sexual motivation and maintained sex hormone secretion homeostasis in CUMS-induced depressed mice. In-depth studies showed that MOO treatment further alleviated sperm morphology and functional defects, demonstrating a novel therapeutic effect of MOO on hypogonadism in depressed mice. These studies elucidate the potential contribution of MOO to the development of hypogonadal symptoms in depressed organisms and provide new insights into the pharmacodynamics of MOO in the prevention and treatment of depression and sexual dysfunction. To better understand the associated brain/reproductive system axis disorders, the underlying mechanisms of sexual dysfunction will be further explored in subsequent studies by combining *in vitro* experiments.

As a human disease supplement or treatment, MOO has certain potential limitations, and the mechanism of action of MOO is not yet very clear. Although there have been some studies exploring the antidepressant mechanism of action of MOO and found that it may work through the regulation of certain metabolic pathways in the gut microbiota, but at present there is not a completely clear understanding of its specific mechanism of action, which may affect the MOO of further application of the study. Taken together, our results have shown that MOO has a dual therapeutic effect on stress-induced depression and stress-induced sexual dysfunction, demonstrating the multimodal effects of MOO. The results of this study provide important data support, broaden behavioral neuroscience insights into the role of MOO, and lay the groundwork for its possible use in the development of potential therapeutic strategies for the treatment of co-occurring depression and sexual dysfunction in the organism.

## Data Availability

The original contributions presented in the study are included in the article/supplementary material, further inquiries can be directed to the corresponding authors.
